# Factors Influencing Smoking Behavior Among Japanese Employees: A Cross-Sectional Study

**DOI:** 10.7759/cureus.78835

**Published:** 2025-02-10

**Authors:** Ashraful Islam Reshad, Hiromi Kawasaki, Md Moshiur Rahman, Misaki Shiraishi, Sae Nakaoka, Sadia Alam Aivey

**Affiliations:** 1 Department of Health Sciences, Graduate School of Biomedical and Health Sciences, Hiroshima University, Hiroshima, JPN

**Keywords:** decision balance, health literacy, self-efficacy, smoker, smoking behavior

## Abstract

Introduction: Globally, smoking causes several health risks, burdening public health and economic productivity. Evaluating employees' smoking cessation tendencies is crucial for companies and the nation. Therefore, this study examines the relationship between sociodemographic factors and three key determinants of smoking cessation: health literacy, decision balance, and self-efficacy among company employees.

Methodology: This cross-sectional study was conducted from October to November 2023 with manufacturing industrial workers in Hiroshima city. All 1,519 company employees were the study participants and were enrolled according to the inclusion and exclusion criteria. The study survey included three individual-developed scales: health literacy, smoking abstinence self-efficacy, and decision balance.

Results: A total of 820 participants responded to the whole survey, and their data were analyzed. Among them, 636 (77.6%) were men and 184 (22.4%) were women with various age groups. The highest number of participants, 151 (18.4%), were working in the Hiroshima Gas Techno-Service, and the smallest, 17 (2.1%), in the Hiroshima Gas Life. Most participants are nonsmokers, 625 (76.2%), while 195 (23.8%) are smokers. The binary logistic regression revealed that female nonsmoker participants (p < 0.001), those in the 20-year-old age group (p = 0.006), and individuals in management positions (p = 0.029) have a statistically significant association with smoking status. No significant difference was found in the mean score on health literacy, while the smoking abstinence self-efficacy scale (p <0.001) had a strong relationship with smoking behavior. Regarding the decision balance scale, the high responses in the pros showed a better statistical significance (p < 0.002), indicating that smokers did not significantly perceive the benefits of decision balance related to smoking.

Conclusion: Understanding smoking behavior and the factors influencing cessation efforts remains a critical public health priority. This study identified age, gender, self-efficacy, and decision balance as key factors associated with smoking cessation tendencies.

## Introduction

Smoking is a major public health concern that negatively affects preventable diseases and disabilities, including overall public health outcomes and economic productivity worldwide. The adverse health effects of smoking are well documented, such as cardiovascular diseases, respiratory illnesses, and various forms of cancer [1,2]. Globally, in 2020, the adult smoking prevalence was 32.6% for men and 6.5% for women, while regularly smoking tobacco caused 7.0 (2-11.2) million deaths [3,4]. In Japan, the prevalence of smoking was 23.5% in 2022,​​ with an increasing trend among adults, especially men [5]. Despite widespread public health campaigns and policy measures aimed at reducing smoking prevalence, the habit persists, particularly among certain sociodemographic groups, particularly in the workplace, where it can lead to decreased productivity and increased healthcare costs. A previous study examines the variations in smoking cessation efforts motivated by health concerns (56.2%), social factors (23.9%), and cost increases (25.8%) [6]. Understanding the factors that influence smoking behavior and cessation efforts is crucial for effective interventions to reduce smoking rates and improve population health.

High smoking prevalence within a company not only diminishes productivity but also adversely impacts employees' health, leading to increased medical costs across various stages of their lives. Smokers have higher absenteeism and overall occupation-related activity impairment compared with former and nonsmokers [7,8]. On the other hand, between 1999 and 2018, the prevalence of secondary smoking exposure decreased in many countries (43.5%); however, until now, it has remained unchanged (35.9%) or increased (20.6%) in other countries [4]. Sequentially, secondhand smoke increases the risk of alcohol dependency, ischemic heart disease, stroke, type 2 diabetes, and lung cancer [9,10]. Moreover, assessing employees' smoking cessation tendencies is a critical concern for individual companies and the nation as a whole. Furthermore, research studies explored the unique cultural, social, and psychological factors that may impact smoking cessation intention. Correspondingly, several factors influence smoking cessation, such as personal and lifestyle, nicotine addiction, and health literacy [11]. According to research, comprehensive health literacy in Japan is comparatively lower than that in Europe [12]. Besides, despite the importance of these psychological factors, there has been limited research on the specific influences that create an outline for smokers' intentions to quit, especially in certain populations. Smoking-related beliefs, such as self-exempting beliefs and self-efficacy, are essential factors that might influence a smoker's decision and intention to quit. Therefore, this study examines the relationship between sociodemographic factors and three key determinants of smoking cessation: health literacy, decision balance, and self-efficacy among company employees. The researcher addressed the operational definitions for those determinants. Health literacy is an individual's ability to understand health information and the capability to collect, use, and classify the information based on health needs. Self-efficacy is an individual’s belief in their ability to control and perform an action to achieve a goal. It is a measure of self-confidence level to control self-behavior. Decision balance is defined as an individual’s expertise in making the right choice and appropriate decisions for better health outcomes. By examining these associations, the study seeks to identify specific sociodemographic groups that may require tailored support and resources to enhance their smoking cessation efforts. The findings will contribute to developing targeted, evidence-based interventions to promote smoking cessation in the workplace, ultimately leading to improved health outcomes and reduced health disparities among employees.

## Materials and methods

Study design, area, and participants

A cross-sectional study was conducted with manufacturing industrial workers in Hiroshima City, Japan.

Sample and data collection

A large gas production and manufacturing company in Hiroshima Prefecture conducted a survey to promote employee empowerment for nonsmoking initiatives and to prevent secondhand smoke exposure. This company was the main investigator for this study, recognized for having the highest sales volume in Hiroshima Prefecture, and was actively involved in sustainable development goals, particularly in areas such as carbon dioxide reduction and carbon neutrality. Thus, the company was highly interested in social issues and involved participation in employee health management, which is recommended in Japan. The survey results were used to plan employee health care by the public health nurse. The data were then transferred to the Hiroshima University for statistical analysis. Therefore, this company agreed to conduct this study among their employees.

The sample population was all employees of the company. The company had a total of 1,519 employees, and a convenience sampling technique was used in this study. All of them were invited to participate in this study, considering their respective sampling technique from the company's health management department. The data collection period was from October 10, 2023, to November 10, 2023. This study used secondary data from an internet- and paper-based survey conducted at the company in Hiroshima, Japan. Furthermore, this survey included three individual-developed scales related to smoking cessation, each with a high Cronbach alpha (0.70-0.83) [13]. Each questionnaire contained questions that were designed by specific authors to assess smoking cessation, and the company believes that those questions were necessary to address smoking cessation (Appendices) [13,14]. Those scales are the health literacy scale (five items), the smoking abstinence self-efficacy questionnaire scale (six items), and the decision balance scale (20 items). All the scales consisted of a five-point Likert scale, and the highest score indicated better outcomes among the participants.

Enrolment

The participants were enrolled in this study according to the inclusion and exclusion criteria. The inclusion criteria specified participants who were willing to participate and provided consent. The participants could join this study in terms of any gender and without any fixed-term working duration or experiences. The participants who did not respond to the whole survey were excluded from the study during the data analysis.

Data analysis

The descriptive analysis was used to analyze the sociodemographic data. The categorical variables were demonstrated in frequency and percentage. The total score of each scale was calculated, and the total score of the scales was analyzed as continuous variables. The total score of the decision-making scale was divided into two groups: pros and cons. Here, each group consisted of 10 questions, while the pros group was related to the question of smoking benefits and the cons related to the question of smoking disadvantages. Then, the binary logistic regression analysis was performed to evaluate the association of smoking cessation with sociodemographic status, health literacy, self-efficacy scale, and decision balance regarding the smoking behavioral status as a dependent variable. This binary logistic regression was performed to predict a binary outcome, considering the dependent variables were categorized into two categories (yes and no). Gender, age, current working place, working position, type of work, health literacy, decision balance, and self-efficacy scale are considered independent variables. The results of logistic regression were presented as odds ratios (ORs). The data were analyzed using the statistical software Statistical Package for the Social Sciences (version 27, IBM Corp., Armonk, NY). A 95% confidence interval (CI) was used to determine statistical significance.

Ethical consideration

The studies involving humans and this study were approved by the Institutional Review Board, Hiroshima University Ethics Committee, Hiroshima, Japan (approval no. E2023-0248). The studies were conducted under local legislation and institutional requirements.

As part of the training aimed at promoting nonsmoking for employees’ health management, a questionnaire was administered to all employees by the company. The questionnaire consisted of several questions; the company believes those questions were necessary to address smoking cessation. For study participation, consent was obtained from the participants, and the responses were voluntary and anonymous. The investigation was conducted anonymously. Data were monitored only by the authorized researchers who were involved in this study and kept in a secure place. During data analysis, an identification number was used without any personal information of the participants. A basic analysis was performed by the healthcare personnel. As a joint research project between the company and Hiroshima University, with the consent of the administrator, the data were provided to Hiroshima University for further detailed analysis. The company does not influence the outcome of the study.

## Results

A total of 820 participants responded to the entire survey, and we analyzed their data in this study. In the sociodemographic characteristics of the participants, we found that most participants were men, 636 (77.6%), while 184 (22.4%) participants were women. The result demonstrated that the age of the participants was aligned into various groups, with the largest group, 329 (40.1%), being 50 years old. In contrast, the smallest group, five (0.6%) of the participants, were teenagers. The participants were employed in several workplaces. The highest number of participants, 151 (18.4%), were working in the Hiroshima Gas Techno-Service and the smallest, 17 (2.1%), in the Hiroshima Gas Life. Among them, 146 (17.8%) were responsible for management positions, 34 (4.1%) were executive officers, and others were engaged in different work positions in the company. All of them were assigned to various types of work categories, particularly in administrative positions 315 (38.4%), involved in outdoor fieldwork 213 (26.0%), sales 139 (17.0%), and indoor fieldwork 52 (6.3%). Regarding the prevalence of smoking status, the majority of participants were nonsmokers, 625 (76.2%), while 195 (23.8%) were smokers (Table 1).

**Table 1:** Sociodemographic characteristics of the participants (n = 820) The option "Other" was applicable for those who were not related in the mentioned options in each variable

Variables	n (%)
Gender	
Male	636 (77.6)
Female	184 (22.4)
Age	
Teens (10-19 years)	5 (0.6)
20 years	98 (12.0)
30 years	86 (10.5)
40 years	183 (22.3)
50 years	329 (40.1)
60 years and above	119 (14.5)
Current work place	
Hiroshima Gas Management Department	104 (12.7)
Hiroshima Gas Energy Business Division	121 (14.8)
Hiroshima Gas Pipeline Business Division	131 (16.0)
Hiroshima Gas Production Business Division	52 (6.3)
Hiroshima Gas Propane	100 (12.2)
Hiroshima Gas Techno-Service	151 (18.4)
Hiroshima Gas Mate	94 (11.5)
Hiroshima Gas Life	50 (6.1)
Other	17 (2.1)
Work position	
Executive officer	34 (4.1)
Management position	146 (17.8)
Other than management and executive officer position	640 (78.0)
Type of work	
Sales position	139 (17.0)
Administrative position	315 (38.4)
Outdoor field work	213 (26.0)
Indoor field work	52 (6.3)
Other	101 (12.3)
Smoking status	
Nonsmoker	625 (76.2)
Smoker	195 (23.8)

Concerning the sociodemographic characteristics, the nonsmoker participants from the female gender (OR: 0.291, 95% CI: 0.156-0.541, p < 0.001) and 20 years old aged group (OR: 0.320, 95% CI: 0.141-0.725, p = 0.006) had a statistically significant association with smoking status compared with male gender and other aged group participants, respectively. Considering the current work position, the management position (OR: 0.298, 95% CI: 0.101-0.883) has a dynamic influence on smoking status, which is statistically significant (p = 0.029). The employees' mean score on the health literacy scale was 17.57 (standard deviation = 2.942), which was higher than the national average in Japan; however, no significant difference was found (Table 2).

**Table 2:** The mean and standard deviation of all three scales (n = 820)

Variables	Mean	Standard deviation
Health literacy scale (total score)	17.57	2.942
Smoking abstinence self-efficacy scale (total score)	23.50	7.205
Decision balance scale		
Cons (total score)	31.70	7.566
Pros (total score)	35.60	7.192

Subsequently, smoking status has a strong significant association (OR: 0.833, 95% CI: 0.805-0.861, p < 0.001) with the smoking abstinence self-efficacy scale. This result demonstrates that the self-efficacy scale had a strong relationship with smoking behavior among the study participants, which was statistically significant. Considering the high score on the self-efficacy scale, smokers had a strong desire for smoking cessation. However, we explored that the smoker participants had less self-efficacy to quit smoking. Then, the smoking status of the participant with the high responses in the pros of the decision balance scale has a better statistical outcome (OR: 0.950, 95% CI: 0.92-0.982, p < 0.002) (Table 3).

**Table 3:** Association of smoking status with sociodemographic status, health literacy, smoking abstinence self-efficacy scale, and decision balance (n = 820) B: logit coefficient; OR: odds ratio; CI: confidence interval Nagelkerke R^2^ = 0.441/Hosmer and Lemeshow test = 0.077

Variables	B	OR (95% CI)	p value
Sociodemographic characteristics			
Gender			
Male	Ref	-	-
Female	-1.236	0.291 (0.156-0.541)	0.000
Age			
60 years and above	Ref	-	-
Teens	-21.053	-	0.999
20 years	-1.140	0.320 (0.141-0.725)	0.006
30 years	-0.398	0.672 (0.31-1.456)	0.313
40 years	-0.286	0.751 (0.388-1.456)	0.397
50 years	-0.521	0.594 (0.322-1.096)	0.095
Current work position			
Executive officer	Ref	-	-
Management position	-1.210	0.298 (0.101-0.883)	0.029
Other than management and executive officer positions	-0.636	0.530 (0.2-1.399)	0.200
Type of work			
Sales and administrative position	Ref	-	-
Outdoor fieldwork	0.223	1.250 (0.773-2.02)	0.363
Indoor fieldwork	-0.289	0.749 (0.333-1.686)	0.485
Others	0.063	1.065 (0.561-2.022)	0.848
All scales			
Health literacy scale (total score)	0.070	1.072 (0.998-1.152)	0.056
Smoking abstinence self-efficacy scale (total score)	-0.183	0.833 (0.805-0.861)	0.000
Decision balance scale			
Cons (total score)	-0.031	0.969 (0.936-1.003)	0.074
Pros (total score)	-0.051	0.950 (0.920-0.982)	0.002

Smokers did not significantly perceive the benefits of decision balance regarding smoking. Instead, it was suggested that nonsmokers were more likely to recognize these benefits. As a result, nonsmokers were not believed to have a strong attachment to or sense of pride in smoking. Therefore, the result illustrates that the participants who were smokers with high self-efficacy did not have undesirable beliefs about smoking, such as smoking is enjoyable, helps in relaxation, and improves mood for enhanced concentration and productivity at work. Thus, smoking behavior has a relationship among male gender, other than younger age, managers, self-efficacy, and perceived benefits of tobacco.

## Discussion

The findings of this study have significant implications for public health practice and policy by identifying the factors of smoking behavior, such as sociodemographics, health literacy, self-efficacy, and decision balance. The study provides valuable insights into the barriers and facilitators of smoking cessation among company employees. These insights can inform the design of more effective, targeted interventions that address different sociodemographic groups' specific needs and challenges. Moreover, the study underscores the importance of self-efficacy as a foundational element in smoking cessation efforts and highlights the need for comprehensive workplace health education programs.

The workplace provides a unique and strategic context for smoking cessation interventions. Employers have a vested interest in promoting the health and well-being of their employees, as smoking is associated with increased absenteeism, reduced productivity, and higher healthcare costs. Workplace-based smoking cessation programs can leverage existing organizational structures and resources to support employees in their quit attempts. Understanding the association of sociodemographic status with health literacy, decision balance, and self-efficacy among company employees can inform the design and implementation of effective workplace interventions.

High levels of health literacy (p = 0.056) have been associated with better health outcomes and more effective management of chronic conditions, including smoking cessation. Conversely, low health literacy can impede an individual's ability to understand health information and make informed decisions about their health [15]. Individuals with higher health literacy are more likely to comprehend the health risks associated with smoking, recognize the benefits of quitting, and navigate the healthcare system to seek cessation support. Conversely, low health literacy can act as a barrier to effective smoking cessation, leading to poorer health outcomes and perpetuating health disparities. This study found no clear relationship between health literacy and smoking cessation. Focusing on this study's aim, no statistically significant relationship was observed between health literacy and smoking cessation. The health literacy score of the subjects surpassed the average of Japan, which is deemed to be the rationale for the absence of a definitive association observed [16]. There is a tendency to support the research so far. Further possible explanations could be limiting the ability to detect meaningful variation in smoking cessation behavior based on health literacy due to the long-term habituation to the smoking of the participants. As a cross-sectional study, this research evaluated the associations at a single point in time, which may not fully reflect the dynamic nature of smoking cessation efforts. Additionally, less enthusiasm to quit smoking and support for accessibility to cessation resources may play a vibrant role in influencing smoking behavior beyond health literacy. Although higher health literacy scores were observed in this sample compared to the national average, the lack of a significant relationship with smoking cessation suggests that other factors, such as self-efficacy and decision balance, might play a more dominant role in cessation outcomes. Thus, future studies incorporating a longitudinal design or including a more diverse sample with a wider range of health literacy levels could provide deeper insights into this relationship.

High self-efficacy is associated with a greater likelihood of attempting to quit and maintaining abstinence [16]. Smoking behavior has been expected to relate closely to an individual's difficulty in controlling the desire to smoke. The findings of this study support this conception, indicating a significant association between smoking behavior and low self-efficacy. Specifically, individuals with lower levels of self-efficacy, or confidence in their ability to resist smoking desire, may find it more challenging to smoke cessation. This relationship suggests that self-efficacy plays a critical role in changing smoking habits. Consequently, enhancing self-efficacy through tailored interventions can significantly improve smoking cessation outcomes. Self-efficacy is closely linked to the experience of prior successes, which reinforces confidence in achieving desired outcomes [17]. For smokers, minimizing mistakes during cessation efforts is crucial. Regular, supportive feedback from a designated individual, such as the primary health care worker who acknowledges progress and encourages continued cessation efforts, may be highly beneficial. This frequent reinforcement of progress can support individuals in staying motivated and committed to quitting smoking. Workplace settings, where individuals spend a significant portion of their time, offer an ideal environment for implementing such interventions to promote sustained smoking cessation.

A favorable decision balance boosts the perceived advantages of improving smoking behavior, which is associated with greater motivation to cease smoking [18]. The study suggests that smokers may have limited awareness of the benefits and disadvantages associated with smoking. Poor awareness may influence their continued smoking behavior because of their less recognition of the negative health impacts or the potential benefits of quitting. Furthermore, smoking behavior is often associated with weak recognition of its benefits, while smokers may not perceive the risks of smoking fully. Accordingly, the decision to quit smoking is less influenced by a clear understanding of its destructive effects. Therefore, there is a high demand for interventions aimed at increasing smokers' awareness regarding the disadvantages of smoking. The ability to collect and classify smoking information is health literacy. It plays a dynamic role in determining decision balance regarding smoking cessation, as it influences an individual's ability to evaluate the risks and benefits of quitting. Individuals with higher health literacy are more likely to comprehend the negative health consequences of smoking, assess the long-term advantages of cessation, and make better decisions based on reliable health information. Conversely, individuals with lower health literacy may struggle to evaluate the pros and cons of decision balance effectively, potentially misjudging the risks of smoking. Thus, implementing strategies that promote a deeper understanding of smoking’s risks, enhance health literacy, and actively support cessation efforts would be beneficial for both individuals and organizations [19]. These approaches represent valuable propositions for the company seeking to reduce employee smoking rates.

Understanding the decision balance in various sociodemographic groups can provide insights into their smoking behaviors and readiness to quit. This study's results exposed that the decision balance requires further investigation in other populations for justification. Previous research has identified several key factors that impact smoking cessation, including sociodemographic characteristics, health literacy, self-efficacy, and decision balance [20-22]. Sociodemographic factors, including age, gender, education level, income, and employment status, have been shown to influence smoking behaviors and cessation outcomes [23]. For instance, smoking prevalence is typically higher among individuals with lower socioeconomic status, lower educational attainment, and certain occupational groups. These factors also affect health literacy, self-efficacy, and decision balance, thereby shaping the overall success of smoking cessation efforts. The limited awareness of smoking's risks and benefits among some employees suggests a need for interventions that focus on enhancing decision balance. Additionally, sociodemographic factors, such as age and socioeconomic status, might influence individuals' awareness and perception of smoking’s harms, and future studies should explore these factors more deeply. The results of this study revealed a significant relationship between an individual's role within the company and their smoking behavior. Therefore, the company should focus on its role along with health and productivity management and prioritize strengthening the following points. Additionally, the findings may contribute to enhancing national and community-level policies by emphasizing the role of tailored health communication, accessibility to cessation resources, and integrating smoking cessation support into primary healthcare services. While the results suggest that self-efficacy and decision balance are associated with smoking cessation, the cross-sectional nature of the study precludes definitive conclusions about causality. Longitudinal studies are needed to establish the directionality of these relationships.

Health literacy regarding smoking cessation is considered the ability to obtain, process, and understand basic health information and services needed to make appropriate health decisions, which plays a pivotal role in smoking cessation. Self-efficacy, the belief in one’s ability to perform specific behaviors in challenging situations, is a critical determinant of smoking cessation. It influences how individuals cope with withdrawal symptoms, manage stress, and resist temptations to smoke. The concept of decision balance, originating from the transtheoretical model of behavior change, refers to the weighing of pros and cons associated with smoking and its cessation. Individuals’ readiness to quit smoking is influenced by their perceived benefits and drawbacks of continuing to smoke versus quitting. Understanding these factors is critical in creating effective cessation programs and policies tailored to high-risk groups.

Limitations

In this study, we have a few limitations. This survey was a cross‑sectional study, and the question items were limited. Therefore, we were not able to collect the data extensively. The data were collected from only one company in the Hiroshima prefecture, which does not represent the entire situation of Japan. This company was active in managing the health of its employees. This study's results showed that most participants were nonsmokers, which may have influenced the data analysis and introduced potential bias. Thus, to minimize the limitations and extend this study's findings, the interventional and longitudinal study within multiple companies in urban and rural areas is recommended in the future.

## Conclusions

Smoking cessation remains a critical public health priority, and understanding the factors influencing cessation efforts is essential for developing effective interventions. This study explored the age and female gender associated with smoking tendencies, providing insights into demographic factors that may influence smoking behavior. Subsequently, no significant difference was exposed in the health literacy scale; however, the smoking abstinence self-efficacy scale had a strong relationship with smoking behavior. It indicates an individual's confidence in their ability to resist smoking. Next, the pros of the decision balance scale had a satisfactory result, which specifies that smokers did not significantly perceive the benefits of decision balance regarding smoking cessation. The insights gained from this research can guide the development of tailored, evidence-based strategies to support smoking cessation in the workplace, ultimately leading to healthier, more productive employees and a reduction in smoking-related health disparities.

## Appendices

Tobacco questionnaire

Q1) Please select your gender.

1. Male

2. Female

3. Nonbinary

Q2) Please select your age group.

1. Teens

2. 20s

3. 30s

4. 40s

5. 50s

6. 60s and above

Q3) Please select the option that best describes your current workplace (as of October 1, 2023).

1. Hiroshima Gas Management Department (Audit Department, Secretary Department, Business Planning Department, General Affairs Department, Environment/Social Contribution Department, Personnel Department, Accounting Department, Materials Department, Resource/Overseas Business Department, Digital Strategy Promotion Department, Technology Research Institute)

2. Hiroshima Gas Energy Business Division (Sales Planning Department, Sales Promotion Department, Customer Department, Sales Technology Department, Household Energy Sales Department, Industrial Energy Sales Department, Business Energy Sales Department, Kure Branch Office Sales Group, Onomichi Branch Office Sales Group)

3. Hiroshima Gas Pipeline Business Division (Supply Equipment Department, Supply Safety Department (including Kure Supply Group and Onomichi Supply Group), Equipment Management Department, Technical Training Center)

4. Hiroshima Gas Production Business Division (Hatsukaichi Plant (including Kaida Base and East Hiroshima Manufacturing), Bingo Plant, Engineering Department)

5. Hiroshima Gas Propane

6. Hiroshima Gas Techno-Service

7. Hiroshima Gas Mate

8. Hiroshima Gas Life

9. Other

Q4) Please select your position.

1. Executive Officer

2. Management Position (Department Head, Section Chief, Manager, Section Manager, Assistant Section Chief)

3. Other than 1 and 2

Q5) Please select your type of work.

1. Sales position

2. Administrative position

3. Outdoor field work

4. Indoor field work

5. Other (Please specify)

Q6) Please select the option that best describes your current smoking status.

1. Never smoked (or smoked only a few times in the past)

2. Quit smoking more than six months ago

3. Quit smoking less than six months ago

4. Currently smoking (have never quit smoking)

5. Intend to quit smoking within one month

6. Intend to quit smoking within more than one month but less than six months

Q7) The following questions (Q7-Q10) are for smokers only.

Please select the numbers that apply to the tobacco product(s) you currently use. *Multiple selections are allowed (for smokers only).

1. Cigarettes

2. Heated tobacco products

3. Electronic cigarettes

4. Other (please specify)

Q8) How many cigarettes do you smoke per day? (for smokers only)

1. 0 cigarettes

2. 1-5 cigarettes

3. 6-10 cigarettes

4. 11-15 cigarettes

5. 16-20 cigarettes

6. 21 or more cigarettes

Q9) How many times per day do you use heated tobacco or electronic cigarettes? (for smokers only)

(1 stick = 1 unit, 1 capsule = 4 units; please calculate the total number)

1. 0 times

2. 1-5 times

3. 6-10 times

4. 11-15 times

5. 16-20 times

6. 21 times or more

Q10) Among the total number of cigarettes you smoke per day, please indicate the percentage that you smoke during work hours. (for smokers only)

1. 0%

2. 1%-4%

3. 5%

4. 6%-9%

5. 10%

Q11) Do you find cigarette smoke from smokers unpleasant?

1. Find it unpleasant

2. Somewhat find it unpleasant

3. Somewhat do not find it unpleasant

4. Do not find it unpleasant

Q12) How do you perceive your own health status?

1. Consider myself very healthy

2. Consider myself relatively healthy

3. Do not consider myself very healthy

4. Do not consider myself healthy

The following questions (Q13-Q17) are about "Information on Health Maintenance and Promotion."

Q13) Can you gather information from various sources such as newspapers, books, television, and the internet?

1. Not at all

2. Not much

3. Neutral

4. Fairly well

5. Very well

Q14) Can you select the information you seek from the abundance of available information?

1. Not at all

2. Not much

3. Neutral

4. Fairly well

5. Very well

Q15) Can you understand information and communicate it to others?

1. Not at all

2. Not much

3. Neutral

4. Fairly well

5. Very well

Q16) Can you determine the reliability of information?

1. Not at all

2. Not much

3. Neutral

4. Fairly well

5. Very well

Q17) Can you make plans and take actions for health improvement based on information?

1. Not at all

2. Not much

3. Neutral

4. Fairly well

5. Very well

The following questions (Q18-Q25) ask about how important you place on the content of items Q18-Q37 for your decision-making regarding smoking.

Q18) Smoking is enjoyable.

For those who have no smoking experience, please imagine how you would feel if you were to smoke and answer.

1. Not important at all

2. Not very important

3. Neutral

4. Somewhat important

5. Very important

Q19) My smoking negatively affects the health of others.

1. Not important at all

2. Not very important

3. Neutral

4. Somewhat important

5. Very important

Q20) I like the look of people who smoke.

1. Not important at all

2. Not very important

3. Neutral

4. Somewhat important

5. Very important

Q21) If I were to become ill due to smoking, it would bother people around me.

1. Not important at all

2. Not very important

3. Neutral

4. Somewhat important

5. Very important

Q22) Smoking helps me relax and improves my mood.

1. Not important at all

2. Not very important

3. Neutral

4. Somewhat important

5. Very important

Q23) Some people think that I lack the judgment to quit smoking.

1. Not important at all

2. Not very important

3. Neutral

4. Somewhat important

5. Very important

Q24) If I try to quit smoking, I will become irritable and lash out at others.

1. Not important at all

2. Not very important

3. Neutral

4. Somewhat important

5. Very important

Q25) Smoking harms my health.

1. Not important at all

2. Not very important

3. Neutral

4. Somewhat important

5. Very important

Q26) People around me think it's better to smoke easily than to struggle with quitting smoking.

1. Not important at all

2. Not very important

3. Neutral

4. Somewhat important

5. Very important

Q27) I feel embarrassed about wanting to smoke.

1. Not important at all

2. Not very important

3. Neutral

4. Somewhat important

5. Very important

Q28) I like myself when I smoke.

1. Not important at all

2. Not very important

3. Neutral

4. Somewhat important

5. Very important

Q29) Second-hand smoke from smoking bothers people around me.

1. Not important at all

2. Not very important

3. Neutral

4. Somewhat important

5. Very important

Q30) Smoking enhances concentration and productivity at work.

1. Not important at all

2. Not very important

3. Neutral

4. Somewhat important

5. Very important

Q31) I feel disdain from others for ignoring warnings about smoking.

1. Not important at all

2. Not very important

3. Neutral

4. Somewhat important

5. Very important

Q32) Smoking relieves tension.

1. Not important at all

2. Not very important

3. Neutral

4. Somewhat important

5. Very important

Q33) People close to me do not appreciate my smoking.

1. Not important at all

2. Not very important

3. Neutral

4. Somewhat important

5. Very important

Q34) I believe that continuing to smoke is my own decision.

1. Not important at all

2. Not very important

3. Neutral

4. Somewhat important

5. Very important

Q35) I consider myself foolish for ignoring warnings about smoking.

1. Not important at all

2. Not very important

3. Neutral

4. Somewhat important

5. Very important

Q36) When I haven't smoked for a while, I feel really good when I do smoke.

1. Not important at all

2. Not very important

3. Neutral

4. Somewhat important

5. Very important

Q37) If I didn't smoke, I would probably feel more energetic now.

1. Not important at all

2. Not very important

3. Neutral

4. Somewhat important

5. Very important

The following questions (Q38-Q40) ask about what you would do if you were offered tobacco, even if you are a nonsmoker or currently do not smoke. Please consider how you would respond.

Q38) When you are feeling unsettled, can you resist smoking?

1. Not at all

2. Not very well

3. Neutral

4. Fairly well

5. Definitely

Q39) When you cannot control feelings of anger, can you resist smoking?

1. Not at all

2. Not very well

3. Neutral

4. Fairly well

5. Definitely

Q40) When using a restaurant or place where smoking is allowed, can you resist smoking?

1. Not at all

2. Not very well

3. Neutral

4. Fairly well

5. Definitely

Q41) Can you resist smoking when you are sad?

1. Not at all

2. Not very well

3. Neutral

4. Fairly well

5. Definitely

Q42) If offered your favorite brand of cigarettes, can you resist smoking?

1. Not at all

2. Not very well

3. Neutral

4. Fairly well

5. Definitely

Q43) When having a conversation while smoking, can you resist smoking?

1. Not at all

2. Not very well

3. Neutral

4. Fairly well

5. Definitely

Q44) Please share any other questions or observations you have.

Thank you for your cooperation.
